# *IGF-1* gene polymorphisms in Polish families with high-grade myopia

**Published:** 2011-09-21

**Authors:** Malgorzata Rydzanicz, Dorota M. Nowak, Justyna A. Karolak, Agata Frajdenberg, Monika Podfigurna-Musielak, Malgorzata Mrugacz, Marzena Gajecka

**Affiliations:** 1 Institute of Human Genetics, Polish Academy of Sciences, Poznan, Poland; 2 Namsos Hospital, Department of Ophthalmology, Namsos, Norway; 3 University Hospital in Linköping, Department of Ophthalmology, Linköping, Sweden; 4 Department of Ophthalmology, Leszno Hospital, Leszno, Poland; 5 Department of Pediatric Ophthalmology, University of Medical Sciences, Bialystok, Poland

## Abstract

**Purpose:**

Recent work has suggested that insulin-like growth factor 1 (*IGF-1*) gene polymorphisms are genetically linked with high-grade myopia (HM), which is a complex-trait eye disorder in which numerous candidate loci and genes are thought to play a role. We investigated whether the *IGF-1* single nucleotide polymorphisms (SNPs) rs6214, rs10860860, and rs2946834 are associated with HM (≤-6.0 diopters [D]) and any myopia (≤-0.5 D) phenotype in Polish families.

**Methods:**

Forty-two multiplex HM Polish families, of whom 127 had HM, participated in the study. All of the family members (n=306) underwent a detailed ophthalmic examination, including axial length measurements. The *IGF-1* SNPs rs6214, rs10860860, and rs2946834 were evaluated by PCR-RFLP and direct sequencing methods. Both Family-Based Association Test (FBAT) and family-based Pedigree Disequilibrium Test (PDT) were used to examine the potential association of the *IGF-1* SNPs rs6214, rs10860860, and rs2946834 with HM or any myopia. To determine the distribution of the HM-associated SNPs rs6214 and rs10860860, 543 unrelated individuals from the general Polish population were also analyzed.

**Results:**

We found no significant association between the *IGF-1* SNPs rs6214, rs10860860, and rs2946834 and HM or any myopia phenotype in Polish HM families. In the general Polish population, the minor allele frequencies of the SNPs rs6214 and rs10860860 did not deviate significantly from the distribution reported for European populations (p=0.629). In the FBAT analysis under the dominant model, the haplotype consisted of T allele of rs10860860, with C allele of rs2946834 of *IGF-1* was found less frequently transmitted to HM individuals (p=0.0065), pointing to a nonassociated or protective haplotype.

**Conclusions:**

Our results do not support recent studies reporting an association of the SNPs rs6214, rs10860860, and rs2946834 in the *IGF-1* gene with HM and any myopia phenotypes. Further replication studies involving other populations are needed to investigate the possible role of *IGF-1* as a potential myopia candidate gene.

## Introduction

Myopia affects 25% of the Western world, making this condition the most common eye disorder in the West and constituting a significant public health and economic problem [1,2]. The cost of optical correction to provide clear distinct vision is considerable. Moreover, the development of high-grade myopia (HM; ≤-6.0 diopters [D]) [3] is a significant risk factor for other ocular diseases, including chorioretinal degeneration, glaucoma, retinal detachment, premature cataracts, and finally blindness [4-6]. Consequently, great efforts have been undertaken to identify and understand the mechanisms underlying the development and progression of myopia.

Myopia has a diverse etiology, with both environmental and genetic factors believed to be involved in the condition’s development and progression. The environmental factors implicated in myopia include near work, light exposure, lack of physical activity, diet, a higher level of education, and urbanization [7-10]. However, HM is highly heritable and often appears as familial ocular disorder, where genetic predisposition seems to be a dominant factor of its development and progression [11-13]. Each type of Mendelian inheritance for familial HM has been described [14,15]. To date, several genetic loci for nonsyndromic myopia (*MYP*) have been mapped, including 12 loci linked to HM: *MYP1*, chromosome Xq28 (OMIM 310460) [16,17]; *MYP2* 18p11.31 (OMIM 160700) [18,19]; *MYP3* 12q21-q23 (OMIM 603221) [20-22]; *MYP4* 7q36 (OMIM 608367) [23]; *MYP5* 17q21-q22 (OMIM 608474) [24]; *MYP11* 4q22-q27 (OMIM 609994) [25]; *MYP12* 2q37.1 (OMIM 609995) [26]; *MYP13* Xq23-q25 (OMIM 300613) [27]; *MYP15* 10q21.1 (OMIM 612717) [28]; *MYP16* 5p15.33-p15.2 (OMIM 612554**)** [29]; *MYP18* 14q22.1-q24.2 (OMIM 255500) [30], and *MYP19* 5p15.1-p13.3 (OMIM 613969) [31]. Moreover, two recent independent genome-wide association studies involving large cohorts of refractive error patients identified loci at chromosome 15q14 and 15q25 [32,33].

Candidate gene association studies have revealed several HM susceptibility genes, including: collagen, type I, alpha 1 (*COL1A1*) [34,35], transforming growth factor, beta 1 (*TGFB1*) [36,37], transforming growth beta-induced factor (*TGIF*) [38,39], lumican (*LUM*) [40,41], hepatocyte growth factor (*HGF*) [42,43], myocilin (*MYOC*) [44,45], paired box 6 (*PAX6*) [46,47], and uromodulin-like 1 (*UMODL1*) [48]. However, positive results have not been replicated, and inconsistent data have been published. Thus, the causative mutation(s) has not yet been found, suggesting genetic heterogeneity among studied populations.

Recently, Metlapally et al. [49] reported a genetic association between the three single nucleotide polymorphism (SNP)s rs6214, rs10860860, and rs2946834 and familial myopia in a large, international cohort of myopia pedigrees of Caucasian origin, suggesting that insulin-like growth factor 1 (*IGF-1*) may be a candidate gene for HM. These three SNPs are located within the *MYP3* locus (OMIM 603221) mapped to chromosomal region 12q21-q23. This locus was previously reported to be associated with autosomal dominant HM [20,21]. The SNP rs6214 (reference allele G) lies in the 3′-untranslated region (UTR) of *IGF-1* (OMIM 147440), whereas the SNPs rs10860860 (reference allele A) and rs2946834 (reference allele C) are located in the noncoding sequence in close proximity to *IGF-1*.

The *IGF-1* gene encodes insulin-like growth factor (pIGF-1), which is a member of the signaling system involved in development, cellular growth, differentiation, protein translation, metabolism, apoptosis, and aging [50,51]. The association of *IGF-1* with numerous human diseases, such as diabetes [52], cancer [53], and growth failure [54] has been reported. *IGF-1* has been also implicated in ocular diseases, including retinopathy of prematurity [55,56], age-related macular degeneration [57], and diabetic retinopathy [58,59]. However, for *IGF-1* to be considered as a candidate gene for HM, previously published findings need to be replicated.

In the present study, we tested the association of rs6214, rs10860860, and rs2946834 in the *IGF-1* gene with HM and any myopia phenotype in 42 Polish families. Our findings do not confirm the results reported previously for another myopic Caucasian cohort, however which was different from the Polish population [49]. To our knowledge, this is the first replication study to screen genetic variants in *IGF-1*, which previous work has suggested may be associated with any myopia and HM phenotypes.

## Methods

### Subjects

Forty-two multigenerational Polish HM families were enrolled in the study. The subjects were classified into three groups, including: 1) affected individuals (HM), 2) individuals with an unknown status, and 3) unaffected persons. All of the affected individuals had: A) bilateral axial HM in excess of or equal to −6.0 D (≤-6.0 D) in at least one eye and in excess of or equal to −5.0 D (≤-5.0 D) in the second eye, B) a history of onset of myopia at age ≤15 years, and C) a multiplex family with affected relatives in different generations. Individuals who were classified as unknown were: A) all children ≤15 years unless they fulfilled criteria for affected status as specified above, or B) individuals who have myopia with −6.0 D< X ≤-4.0 D, or C) individuals with a refractive error of ≤-6.0 D for one eye and a refractive error >-5.0 D for the second eye, or D) individuals with late age of onset (>15 years). All of the remaining individuals were considered unaffected, including hyperopic, normal seeing, and myopic subjects with a spherical refractive error (SPH) in the range from ≤-0.5 D to >-4.0 D.

The SNPs rs6214, rs10860860, and rs2946834 were examined in 306 subjects, including 127 with HM, 148 unaffected, and 31 individuals with an unknown status. In addition, to determine the distribution of the genotypes possibly associated with HM (rs6214 and rs10860860) [49] in the general Polish population, 543 unrelated white Caucasian individuals were examined. The population samples were collected randomly and anonymously, and the myopia status was not determined [60].

All of the individuals (affected, unaffected, and unknown) who participated in this study underwent a complete ophthalmic assessment, including a visual acuity testing, best-corrected visual acuity testing, a slit lamp evaluation, intraocular pressure examination, fundoscopy, axial length determination, keratometry, and refractometry. The detailed clinical evaluation and pedigrees of Polish HM families have been previously described [61]. The clinical characteristics of all of the individuals examined are shown in brief in Table 1. The SPH was used to assign HM status.

**Table 1:** Clinical characteristic of genotyped individuals.

**Clinical characteristic **	**Affected**			**Unaffected**			**Unknown**			**General population group**
No. of individuals	127			148			31			543
**Age at Examination (year)**										
Range	5–87			3–86			3–81			3–83
Mean age (±SD)	40.2 (±20.43)			38.6 (±18.54)			27.1 (±22.63)			41.8 (±16.1)
**Age of Onset (year)**										
Range	2–15			…			…			…
Mean age (±SD)	8.21 (±3.40)									
**Gender**										
Female	77 (60.6%)			79 (53.4%)			13 (41.9%)			285 (52.5%)
Male	50 (39.4%)			69 (46.6%)			18 (58.1%)			258 (47.5%)
**Spherical Refractive Error [D]**	OD	OS	OD+OS	OD	OS	OD+OS	OD	OS	OD+OS	not determined
Mean	−9.34	−9.29	−9.32	−0.05	0.00	−0.03	−2.74	−2.77	−2.75	
(±SD)	(±3.95)	(±3.84)	(±3.89)	(±1.24)	(±1.28)	(±1.26)	(±1.95)	(±2.09)	(±2.00)	
**Cylindrical Refractive Error [D]**	OD	OS	OD+OS	OD	OS	OD+OS	OD	OS	OD+OS	not determined
Mean	−0.90	−0.84	−0.87	−0.39	−0.36	−0.38	−0.29	−0.19	−0.25	
(±SD)	(±1.34)	(±1.15)	(±1.24)	(±0.56)	(±0.65)	(±0.60)	(±0.38)	(±0.46)	(±0.42)	
**Spherical Equivalent [D]**	OD	OS	OD+OS	OD	OS	OD+OS	OD	OS	OD+OS	not determined
Mean	−9.72	−9.63	−9.69	−0.26	−0.17	−0.22	−2.88	−2.87	−2.87	
(±SD)	(±4.09)	(±3.91)	(±4.00)	(±1.19)	(±1.24)	(±1.21)	(±1.95)	(±2.12)	(±2.00)	
**Axial Length [mm]**	OD	OS	OD+OS	OD	OS	OD+OS	OD	OS	OD+OS	not determined
Mean	27.26	27.27	27.27	23.38	23.40	23.39	24.34	23.56	23.95	
(±SD)	(±2.09)	(±1.96)	(±2.03)	(±0.79)	(±0.84)	(±0.82)	(±1.16)	(±4.16)	(±3.05)	

In addition, any myopia phenotype status was established in reference to the criteria published by Metlapally et al. [49], who defined any myopia phenotype as ≤-0.5 D based on SPH. The clinical information data for this category are shown in Appendix 1.

The research protocol was approved by the Institutional Review Board at Poznan University of Medical Sciences in Poland. In accordance with the Declaration of Helsinki, written informed consent was obtained for the genetic studies from all of the participating family members and individuals from the general Polish population group.

### Genotyping of *IGF-1* SNPs rs6214, rs10860860, and rs2946834

The SNPs rs6214 and rs10860860 were analyzed by PCR-RFLP and direct sequencing methods. Two pairs of primers were used to amplify DNA harboring the analyzed SNPs. The amplicons were digested by the restriction enzymes TaiI and NdeI, respectively, according to the manufacturer’s instructions (Fermentas, Vilnius, Lithuania). The detailed PCR-RFLP protocols used in this study are provided in Appendix 2. In addition, all homozygous rs6214 AA and rs10860860 TT samples were verified by direct sequencing using the BigDye® Terminator v3.1 Cycle Sequencing Kit (Applied Biosystems [ABI], Foster City, CA) and an ABI 3730xl analyzer. The results were analyzed by Sequencher® Software 4.10.1 (Gene Codes Corporation, Ann. Arbor, MI). Due to a lack of a restriction site for SNP rs2946834, we analyzed this polymorphism by direct sequencing as described above. The detailed PCR protocol, as well as the primer sequences, are given in Appendix 2.

All of the primer pairs used in this study were designed with the Primer3 v.0.4.0 tool and synthesized by Genomed Co. (Warsaw, Poland).

### Statistical analyses

PEDCHECK version 1.1 [62] was used to determine Mendelian inconsistencies within families. Plink software [63] was used to examine the genotype distribution of the SNPs tested for departures from expectations of the Hardy–Weinberg equilibrium (HWE).

Haploview [64] was used to estimate the linkage disequilibrium (LD) pattern of the analyzed SNPs in the Polish HM families and compare with data pertaining to the CEU population (HapMap Public Release #27).

We tested for an association between the rs6214, rs10860860, and rs2946834 polymorphisms of the *IGF-1* gene and HM and any myopia using the Family-Based Association Test (FBAT, version 1.7.2). FBAT is a generalized approach derived from the transmission/disequilibrium test (TDT) [65], which allows the genotype distribution observed in the affected offspring and the expected distribution to be evaluated. The models of inheritance examined by FBAT included the additive, dominant, and recessive models [66,67]. The FBAT analysis was performed based on the compound null hypothesis of no linkage and no association between the phenotype and the genetic variant. To analyze the haplotype using FBAT, 42 multiplex Polish families were divided into 122 nuclear families (two parents and their offspring), and they were tested using a two-stage procedure. First, the SNPs rs6214, rs10860860, and rs2946834 were examined separately. Testing was then undertaken for possible haplotypes of the specific alleles of rs6214, rs10860860, and rs2946834. Analyses in which there were less than ten informative families for a particular marker were excluded from consideration. Unaffected individuals were included in the study to increase the statistical power of the FBAT analysis.

In addition, to verify the FBAT results, the genetic association between the three selected polymorphisms of the *IGF-1* gene and HM and any myopia phenotypes were tested using the family-based Pedigree Disequilibrium Test (PDT, version 5.1) [68] as described by Metlapally et al. [49]. The PDT analysis program evaluates evidence of LD in general pedigree data.

## Results

The genotype distribution and allele frequencies in affected, unaffected, and individuals with unknown status, as well as in the general Polish population group, are shown in Table 2. All genotypes of examined SNPs were tested for HWE, and no significant deviations were found.

**Table 2:** Genotype distribution and allele frequency of *IGF-1* gene SNPs rs6214, rs10860860, and rs2946834.

		**High-grade myopia**						**Any myopia**							
		**Affected**		**Unaffected**		**Unknown**		**Affected**		**Unaffected**		**Unknown**		**Population group**	
**SNP**	**SNP genotype/allele**	n	%	n	%	n	%	n	%	n	%	n	%	n	%
rs6214	GG	51	40.1	54	36.5	13	41.9	82	39.6	27	34.6	9	42.9	209	38.5
	GA	59	46.5	78	52.7	13	41.9	98	47.3	43	55.1	9	42.9	264	48.6
	AA	17	13.4	16	10.2	5	16.2	27	13.1	8	10.3	3	14.2	70	12.9
	G	161	63.4	186	62.8	39	62.9	262	63.3	97	62.2	27	64.3	682	62.8
	A	93	36.6	110	37.2	23	37.1	152	36.7	59	37.8	15	35.7	404	37.2
rs10860860	AA	57	44.9	60	40.5	15	48.4	96	46.4	23	29.5	13	61.9	248	45.7
	AT	54	42.5	75	50.7	14	45.2	88	42.5	48	61.5	7	33.3	251	46.2
	TT	16	12.6	13	8.8	2	6.4	23	11.1	7	9.0	1	4.8	44	8.1
	A	168	66.1	195	65.9	44	71.0	280	67.6	94	60.3	33	78.6	747	68.8
	T	86	33.9	101	34.1	18	29.0	134	32.4	62	39.7	9	21.4	339	31.2
rs2946834	CC	61	48.8	72	49.0	14	45.2	109	53.4	29	37.2	9	42.9	-	-
	CT	48	38.4	61	41.5	14	45.2	78	38.2	37	47.4	8	38.1	-	-
	TT	16	12.8	14	9.5	3	9.7	17	8.3	12	15.4	4	19.0	-	-
	C	170	68	205	69.7	42	67.7	296	72.5	95	60.9	26	61.9	-	-
	T	80	32	89	30.3	20	32.3	112	27.5	61	39.1	16	38.1	-	-
rs6214/ rs10860860	GG / AA	35	27.6	40	27.0	9	29.0	61	29.5	15	19.2	8	38.1	154	28.4
	GG / AT	13	10.2	13	8.8	4	12.9	18	8.7	11	14.1	1	4.8	53	9.8
	GG / TT	3	2.4	1	0.7	0	0.0	3	1.4	1	1.3	0	0	2	0.4
	GA / AA	19	14.9	19	12.8	5	16.1	31	15.0	7	9.0	5	23.8	81	14.9
	GA / AT	34	26.8	55	37.2	8	25.8	60	29.0	33	42.3	4	19.0	158	29.0
	GA / TT	6	4.7	4	2.7	0	0.0	7	3.4	3	3.8	0	0	25	4.6
	AA / AA	3	2.4	1	0.7	1	3.2	4	1.9	1	1.3	0	0	13	2.4
	AA / AT	7	5.5	7	4.7	2	6.5	10	4.8	4	5.2	2	9.5	40	7.4
	AA / TT	7	5.5	8	5.4	2	6.5	13	6.3	3	3.8	1	4.8	17	3.1
rs2946834/ rs6214	CT / GG	18	14.4	18	12.2	6	19.4	28	13.7	12	15.4	2	9.5	-	-
	CT / GA	24	19.2	32	21.8	7	22.6	41	20.1	18	23.1	4	19.0	-	-
	CC / AA	9	7.2	5	3.4	4	12.9	16	7.8	1	1.3	1	4.8	-	-
	TT / AA	2	1.6	0	0.0	0	0.0	2	1.0	0	0.0	0	0.0	-	-
	CT / AA	6	4.8	11	7.5	1	3.2	9	4.4	7	9.0	2	9.5	-	-
	CC / GA	27	21.6	37	25.2	5	16.1	48	23.5	18	23.1	3	14.3	-	-
	CC / GG	25	20	30	20.4	5	16.1	45	22.1	10	12.8	5	23.8	-	-
	TT / GA	7	5.6	9	6.1	1	3.2	8	3.9	7	9.0	2	9.5	-	-
	TT / GG	7	5.6	5	3.4	2	6.5	7	3.4	5	6.4	2	9.5	-	-
rs10860860/ rs2946834	AT / CT	29	23.2	42	28.6	5	16.1	43	21.1	29	37.2	4	19.0	-	-
	TT / TT	8	6.4	3	2.0	0	0.0	8	3.9	3	3.8	0	0.0	-	-
	TT / CT	5	4	7	4.8	1	3.2	9	4.4	4	5.1	0	0.0	-	-
	AT / TT	7	5.6	6	4.1	3	9.7	8	3.9	6	7.7	2	9.5	-	-
	AA / CC	42	33.6	42	28.6	7	22.6	68	33.3	16	20.5	7	33.3	-	-
	AA / TT	1	0.8	5	3.4	0	0.0	1	0.5	3	3.8	2	9.5	-	-
	AA / CT	14	11.2	12	8.2	8	25.8	26	12.7	4	5.1	4	19.0	-	-
	AT / CC	16	12.8	27	18.4	6	19.4	35	17.2	13	16.7	1	4.8	-	-
	TT / CC	3	2.4	3	2.0	1	3.2	6	2.9	0	0.0	1	4.8	-	-
rs10860860/ rs2946834/ rs6214	AA / CC / AA	3	2.4	1	0.7	1	3.2	4	2.0	1	1.3	0	0.0	-	-
	AA / CC / GA	15	12.0	13	8.8	1	3.2	21	10.3	6	7.7	2	9.5	-	-
	AA / CC / GG	24	19.2	28	19.0	5	16.1	43	21.1	9	11.5	5	23.8	-	-
	AA / CT / GA	4	3.2	4	2.7	4	12.9	10	4.9	0	0.0	2	9.5	-	-
	AA / CT / GG	10	8.0	8	5.4	4	12.9	16	7.8	4	5.1	2	9.5	-	-
	AA / TT / GA	0	0.0	2	1.4	0	0.0	0	0.0	1	1.3	1	4.8	-	-
	AA / TT / GG	1	0.8	3	2.0	0	0.0	1	0.5	2	2.6	1	4.8	-	-
	AT / CC / AA	3	2.4	1	0.7	2	6.5	6	2.9	0	0.0	0	0.0	-	-
	AT / CC / GA	12	9.6	24	16.3	4	12.9	27	13.2	12	15.4	1	4.8	-	-
	AT / CC / GG	1	0.8	2	1.4	0	0.0	2	1.0	1	1.3	0	0.0	-	-
	AT / CT / AA	3	2.4	6	4.1	0	0.0	3	1.5	4	5.1	2	9.5	-	-
	AT / CT / GA	18	14.4	26	17.7	3	9.7	28	13.7	17	21.8	2	9.5	-	-
	AT / CT / GG	8	6.4	10	6.8	2	6.5	12	5.9	8	10.3	0	0.0	-	-
	AT / TT / AA	1	0.8	0	0.0	0	0.0	1	0.5	0	0.0	0	0.0	-	-
	AT / TT / GA	3	2.4	5	3.4	1	3.2	4	2.0	4	5.1	1	4.8	-	-
	AT / TT / GG	3	2.4	1	0.7	2	6.5	3	1.5	2	2.6	1	4.8	-	-
	TT / CC / AA	3	2.4	3	2.0	1	3.2	6	2.9	0	0.0	0	0.0	-	-
	TT / CT / AA	3	2.4	5	3.4	1	3.2	6	2.9	3	3.8	0	0.0	-	-
	TT / CT / GA	2	1.6	2	1.4	0	0.0	3	1.5	1	1.3	0	0.0	-	-
	TT / TT / AA	1	0.8	0	0.0	0	0.0	1	0.5	0	0.0	1	4.8	-	-
	TT / TT / GA	4	3.2	2	1.4	0	0.0	4	2.0	2	2.6	0	0.0	-	-
	TT / TT / GG	3	2.4	1	0.7	0	0.0	3	1.5	1	1.3	0	0.0	-	-

The minor allele frequencies estimated for the general Polish population group were 0.372 and 0.312 for rs6214 A and rs10860860 T, respectively. The coexistence of two minor homozygous genotypes of rs6214 AA and rs10860860 TT was observed in 3.1% of individuals (Table 2). In HM families, the minor allele frequencies for the SNPs analyzed were 0.366, 0.339, and 0.320 for rs6214 A, rs10860860 T, and rs2946834 T, respectively, for HM and 0.367, 0.324, and 0.275, respectively, for any myopia phenotype (Table 2). In the HM category, the coexistence of two minor homozygous genotypes of the SNPs rs6214 AA and rs10860860 TT was observed in 5.5% and 5.4% of affected and unaffected subjects, respectively. The coexistence of two minor homozygous genotypes of rs6214 AA and rs2946834 TT was 1.6% and 0%, whereas, the coexistence of rs10860860 TT and rs2946834 TT, was observed in 6.4% of affected and 2.0% of unaffected subjects, respectively (Table 2). The coexistence of three minor genotypes of the SNPs rs6214 AA, rs10860860 TT, and rs2946834 TT analyzed was found in 0.8% of affected individuals and was absent in unaffected subjects (Table 2).

**Figure 1:**
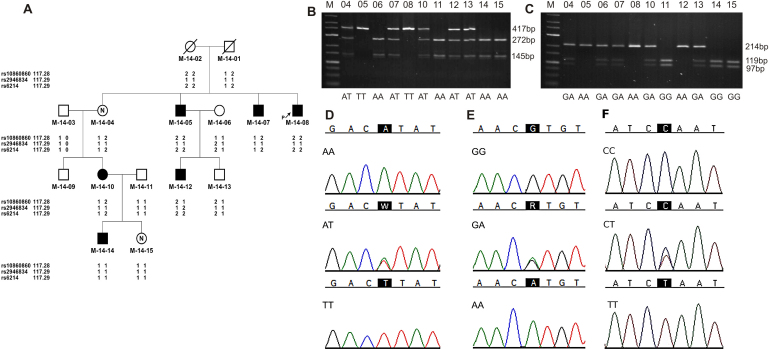
Example of genotype distribution of SNPs rs10860860, rs2946834, and rs6214 in the *IGF-1* gene. **A**: Pedigree of HM-14 family. Filled symbols: individuals with high myopia; open symbols: unaffected individuals; symbols with a question mark: individuals with an unknown disease status; an arrow indicates a proband. Detailed clinical findings in family HM-14 are shown in Appendix 3. **B**: RFLP analysis of SNP rs10860860 and **C**: RFLP analysis of SNP rs6214. Line M: DNA ladder 25–700 bp, remaining lines: numbers of genotyped individuals according to the pedigree HM-14. Below, genotypes are shown. **D**: Partial sequence chromatograms showing rs10860860, **E**: rs6214, and **F**: rs2946834 genotypes, respectively. Black squares indicate SNP alleles.

The FBAT and PDT analyses revealed no significant association between the rs6214, rs10860860, and rs2946834 SNPs analyzed and HM, as well as no significant association with any myopia phenotype. The haplotype consisted of alleles T of rs10860860, and alleles C of rs2946834 of *IGF-1* was found less frequently in HM individuals than expected (p=0.0065). Table 3 presents the Z scores and p values for the tested *IGF-1* polymorphisms and the haplotypes. Moreover, we found a random distribution of genotypes and alleles of the SNPs examined in the Polish HM families (Figure 1 and Appendix 3).

**Table 3:** Family-based association test analyses (FBAT) and pedigree disequilibrium test (PDT) analyses of the *IGF-1* gene SNPs rs6214, rs2946834, and rs10860860.

**High-Grade Myopia**									
		**FBAT**							
		**additive**		**dominant**		**recesive**		**PDT**	
**SNP**	**Allele**	**Z**	**p**	**Z**	**p**	**Z**	**p**	**Z**	**p**
rs10860860	T	−0.781	0.434947	−0.946	0.344327	−0.137	0.890758	−1.068	0.2855
	A	0.781	0.434947	0.137	0.890758	0.946	0.344327	1.068	0.2855
rs2946834	T	0.899	0.368809	0.454	0.649731	1.044	0.296272	0.697	0.4855
	C	−0.899	0.368809	−1.044	0.296272	−0.454	0.649731	−0.697	0.4855
rs6214	A	−0.052	0.958800	0.234	0.815084	−0.412	0.680280	−0.688	0.4913
	G	0.052	0.958800	0.412	0.680280	−0.234	0.815084	0.688	0.4913
rs10860860/ rs6214	AG	−0.347	0.728871	0.340	0.733820	−0.807	0.419469	-	-
	TA	−0.964	0.335015	−0.812	0.416977	-	-	-	-
	AA	1.345	0.178669	1.606	0.108337	-	-	-	-
	TG	−0.003	0.997942	0.308	0.758358	-	-	-	-
rs10860860/ rs2946834	AC	−0.016	0.987072	−0.886	0.375848	0.760	0.447395	-	-
	TT	0.667	0.504478	0.695	0.487210	-	-	-	-
	TC	−2.331	0.019747	−2.723	0.006470	-	-	-	-
	AT	1.898	0.057721	1.898	0.057721	-	-	-	-
rs2946834/ rs6214	CG	−1.472	0.141034	−1.464	0.143106	−0.662	0.508095	-	-
	CA	−0.364	0.715703	−0.906	0.364933	-	-	-	-
	TG	1.172	0.241160	1.358	0.174527	-	-	-	-
	TA	1.433	0.151778	1.433	0.151778	-	-	-	-
rs10860860/ rs2946834/ rs6214	ACG	−1.467	0.142408	−1.425	0.154041	−0.698	0.484867	-	-
	TCA	−2.145	0.031928	−2.528	0.011472	-	-	-	-
	ACA	1.743	0.081412	1.781	0.074903	-	-	-	-
	ATG	1.449	0.147280	1.449	0.147280	-	-	-	-
	TTA	0.830	0.406764	0.830	0.406764	-	-	-	-
	TTG	0.438	0.661696	0.826	0.409048	-	-	-	-
	ATA	-	-	-	-	-	-	-	-
	TCG	-	-	-	-	-	-	-	-
**Any Myopia**									
		**FBAT**							
		**additive**		**dominant**		**recesive**		**PDT**	
**SNP**	**Allele**	**Z**	**p**	**Z**	**p**	**Z**	**p**	**Z**	**p**
rs10860860	T	−1.243	0.213893	−0.636	0.525020	−1.423	0.154872	−1.572	0.1159
	A	1.243	0.213893	1.423	0.154872	0.636	0.525020	1.572	0.1159
rs2946834	T	−0.757	0.449004	−0.850	0.395113	−0.199	0.842542	−0.983	0.3258
	C	0.757	0.449004	0.199	0.842542	0.850	0.395113	0.983	0.3258
rs6214	A	−0.346	0.729034	0.407	0.684086	−1.063	0.287596	−0.567	0.5708
	G	0.346	0.729034	1.063	0.287596	−0.407	0.684086	0.567	0.5708
rs10860860/ rs6214	AG	0.618	0.536845	1.353	0.176003	−0.393	0.694038	-	-
	TA	−0.769	0.441736	−0.362	0.717451	-	-	-	-
	AA	0.794	0.427175	1.252	0.210729	-	-	-	-
	TG	−1.093	0.274437	−0.829	0.407052	-	-	-	-
rs10860860/ rs2946834	AC	0.801	0.423129	0.813	0.416233	0.471	0.637682	-	-
	TT	−0.737	0.460854	−0.432	0.665463	-	-	-	-
	TC	−0.973	0.330541	−1.256	0.209270	-	-	-	-
	AT	1.211	0.226036	1.405	0.160003	-	-	-	-
rs2946834/ rs6214	CG	−0.672	0.501768	−0.81	0.417700	−0.201	0.840646	-	-
	TG	0.276	0.782902	−0.421	0.673876	-	-	-	-
	CA	0.526	0.59913	0.88	0.379065	-	-	-	-
	TA	0.1	0.920244	0.325	0.745094	-	-	-	-
rs10860860/ rs2946834/ rs6214	ACG	−0.270	0.786807	−0.552	0.580836	0.162	0.871179	-	-
	TCA	−0.539	0.590146	−0.902	0.367010	-	-	-	-
	ACA	1.245	0.213008	1.122	0.261914	-	-	-	-
	ATG	1.027	0.304204	1.251	0.211033	-	-	-	-
	TTA	−0.385	0.700077	−0.179	0.857572	-	-	-	-
	TTG	−0.474	0.635663	−0.111	0.911560	-	-	-	-
	ATA	-	-	-	-	-	-	-	-
	TCG	-	-	-	-	-	-	-	-

The analysis of LD between rs6214, rs2946834, and rs10860860 did not reveal high LD between SNP pairs in the Polish HM families. Moreover, the r^2^ values for pairs of markers were similar in Polish HM families and the CEU population (p=0.629).

## Discussion

Genetic association studies, including case-control and family-base studies, have been widely used to search for genetic factors involved in human diseases, including HM [69,70]. Recently, Metlapally et al. [49] suggested that *IGF-1* may be a candidate gene for familial myopia based on a positive association between the SNPs rs6214 and rs10860860 and the HM phenotype and the SNPs rs6214, rs10860860, and rs2946834 and any myopia phenotype.

The SNP rs6214 is considered to be a functional polymorphism due to its location in the 3′-UTR of the *IGF-1* gene. The 3′-UTR, a noncoding sequence, contains regulatory motifs crucial for gene expression, mRNA stability, and cellular location of mRNA or the binding of microRNA [71,72]. Previous work has suggested that sequence changes in this region may alter mRNA stability and lead to altered binding activity to microRNAs, which might downregulate gene expression by mRNA cleavage or translational repression [73,74].

The chick model of experimental myopia suggests a possible role for *IGF-1* in eye growth and elongation; and therefore, in myopia development and progression. Insulin and IGF-1 injected into chick eyes resulted in an increase in the rate of axial length, ocular elongation, and the anterior chamber depth [75,76]. However, these findings do not entirely correspond with published data for the mammal model, with IGF-1 reported to be a weak factor for mouse lens epithelial cell differentiation and proliferation [77,78].

Cordian et al. [9] stated that myopia may be related to impaired metabolic control. They observed that enhanced scleral growth may result from increased levels of insulin and insulin-like growth hormones. Compared with untreated patients with Laron syndrome (LS), susceptibility to mild myopia has been observed in patients with LS (OMIM 262500) who received treatment with IGF-1. However, there was no difference in axial length between IGF-1 treated LS patients and healthy controls [79].

In the present study, we investigated the previously reported association of the SNPs rs6214, rs10860860, and rs2946834 in the *IGF-1* gene with familial HM or any myopia phenotypes. The SNPs selected for the analysis were not in LD in the Polish families; the r^2^ values for pairs of markers were similar in the CEU population and in the Polish HM families. Simultaneously, we estimated the allele distributions of the selected SNPs (rs6214 and rs10860860) in the general Polish population group. We found no significant differences in minor allele frequencies observed in the general Polish population (rs6214 A and rs10860860 T, 0.372 and 0.312, respectively) and the data provided by HapMap Public Release #28 for CEU: Utah residents with Northern and Western European ancestry from the Centre d”Etude du Polymorphisme Humain (CEPH) collection (rs6214 A and rs10860860 T, 0.425 and 0.350, respectively). In our family-based study, we found no evidence of an association between the SNPs rs6214, rs10860860, and rs2946834 in the *IGF-1* gene and any myopia and HM. In contrast to previously published data [49], random distribution of genotypes and alleles were observed in the Polish HM families that we examined. However, the haplotype consisted of allele T of rs10860860, and allele C of rs2946834 of *IGF-1* was found less frequently in HM individuals than expected (p=0.0065), pointing to a protective effect of the haplotype. These and other allele haplotypes were not examined in the Metlapally et al. [49] study.

Over the years, various studies have reported associations between numerous nucleotide variants in several genes and HM [30,42,44,80]. However, subsequent studies have failed to confirm and replicate these associations [40,45,81,82]. Possible reasons for the observed discrepancies in the published HM data, especially in relation to complex genetic traits, include (i) ethnic differences, (ii) sample size, (iii) subject misclassification, (iv) power of association analyses, and (v) criteria for statistical significance [83-85].

We analyzed a homogenous ethnic group. All of the participants were Polish, white Caucasian, and of European origin. Metlapally et al. [49] examined *IGF-1* polymorphisms in a large cohort of Caucasian HM families, but the subjects were derived from different countries, and the majority of families in their study were from the US (60%). European-Americans are often treated as a homogeneous group; however, due to historical immigration, this cohort is formed of diverse source populations [86].

Unlike the study by Metlapally et al. [49], we employed distinct criteria to determine subjects’ HM status. Metlapally et al. [49] based the HM state on SPH, as well as on spherical equivalent (SE) error of −5.00 D or more in at least one eye. We used more stringent criteria in our study. Subjects were classified as affected based on SPH only and a −6.00 D or more in at least one eye and −5.00 D in the second eye in accordance with HM criteria described elsewhere [3]. In some cases, subjects classified as having an unknown myopia status in our study would be classified as having HM data published by Metlapally et al. [49]. Moreover, in our study, individuals with any myopia who did fulfill the criteria for HM or unknown status were treated as unaffected individuals. Metlapally et al. [49] found a significant association between HM and the SNPs rs6214 and rs10860860, as well as any myopia (including low and medium myopia) and the SNPs rs6214, rs10860860, and rs2946834, out of 13 tested SNPs in *IGF-1*. Therefore, we performed an additional analysis using the any myopia phenotype criteria described by Metlapally et al. [49]. Again, the FBAT and PDT analyses showed no positive association between the *IGF-1* SNPs rs6214, rs10860860, and rs2946834 and any myopia phenotype.

Metlapally et al. [49] defined the statistical significance as p≤0.0038, which is in the range where false-positive results are common [84]. Another study has proposed that p≤5×10^−5^ or even p≤2×10^−7^ provides a ratio of a truly positive, reproducible association for candidate genes [87]. Although association studies have identified many putative disease genes, these have often been difficult to confirm. According to Manly [87], irreproducibility might be a consequence of weak statistical power of the original work. Manley’s Better Association for Disease and Gene (BADGE) classification suggests that first-class (p≤2×10^−7^) and second-class (5×10^−6^) associations provide some assurance of reproducibility. However, the reliability of second-class associations depends on assumptions. As these criteria are not fulfilled in the Metlapally et al. [49] study, theoretically, their results will be difficult to replicate in subsequent studies. It is possible that the use of more stringent statistic criteria for positive genetic association [88,89] might yield different study findings.

In conclusion, our study revealed no evidence to support the previously reported genetic association of the *IGF-1* gene polymorphisms rs6214, rs10860860, and rs2946834 with HM and any myopia phenotypes in Polish families.

## Appendix

Appendix 1. Clinical information data for any myopia state. To access the data, click or select the words “Appendix 1.” This will initiate the download of a compressed (pdf) archive that contains the file.

Appendix 2. The PCR and PCR-RFLP conditions for SNPs rs6214, rs10860860, and rs2946834 analyses. *The SNP rs2946834 genotypes were determined by direct sequencing. Due to a lack of a restriction site for rs2946834, the PCR-RFLP assay was not performed. To access the data, click or select the words “Appendix 2.” This will initiate the download of a compressed (pdf) archive that contains the file.

Appendix 3. Detailed clinical findings in family HM-14. *****SPH- spherical refractive error, **†**CYL - cylindrical refractive error. To access the data, click or select the words “Appendix 3.” This will initiate the download of a compressed (pdf) archive that contains the file.
